# Analysis of the use of virtual reality scenarios in education for the treatment of socio-emotional problems

**DOI:** 10.3389/fpsyg.2025.1681128

**Published:** 2026-01-20

**Authors:** René Rodríguez Zamora, Leonor Antonia Espinoza Núñez

**Affiliations:** Facultad de Psicología, Universidad Autónoma de Sinaloa, Mazatlán, Mexico

**Keywords:** virtual reality, higher education, socio-emotional skills, emotional well-being, virtual environments

## Abstract

**Introduction:**

Educational systems have traditionally prioritized disciplinary knowledge, often neglecting students’ socio-emotional development. Recent technological advances and increasing mental health concerns among university students have highlighted the need for innovative educational strategies that integrate emotional learning with immersive technologies such as virtual reality (VR).

**Methods:**

This exploratory quantitative study analyzed the use of virtual reality as a pedagogical strategy for enhancing socio-emotional skills. Two structured self-report surveys were administered to a purposive sample of 14 first-year engineering students at a higher education institution. The variables assessed included perceptual issues, experiences with psychological care, manifestations of emotional distress, family environment, specific emotional-management situations, and academic motivation. Based on students’ responses, suitable virtual reality scenarios were mapped to support the design of individualized educational interventions.

**Results:**

The study identified which VR scenarios students perceived as more helpful for addressing emotional challenges such as anxiety, impulsivity, and communication difficulties. The findings highlight the need to strengthen socio-emotional skill development in higher education, given the wide range of factors that influence students’ emotional well-being and life skills—competencies as essential as disciplinary knowledge throughout their academic trajectory.

**Discussion:**

The results suggest that VR-based educational strategies may offer a feasible and acceptable approach for supporting socio-emotional learning in higher education contexts. Although limited by a small sample size, this exploratory study provides preliminary evidence supporting the integration of immersive technologies into educational settings to foster students’ socio-emotional development.

## Introduction

Educational systems have historically centered their curricula on the development of disciplinary knowledge, often overlooking the emotional and attitudinal dimensions of student formation. However, recent shifts in educational priorities—driven by rapid technological change, increasing mental health concerns among youth, and global disruptions such as the COVID-19 pandemic—have brought the importance of socio-emotional competencies to the forefront (OECD, 2021; UNESCO, 2021).

In recent years, educational environments have undergone rapid technological transformation. The increasing virtualization of social and academic interactions has encouraged educators to seek immersive tools that support both cognitive and emotional development. Virtual reality (VR) has emerged as a promising medium for experiential learning, allowing students to engage in realistic, emotionally charged scenarios in a safe context (Radianti et al., 2020; Parong and Mayer, 2018; Makransky and Petersen, 2021).

The motivation for the present study stems from the growing need to integrate socio-emotional education with technological innovation. By exploring VR as a pedagogical resource for emotional self-regulation, this research contributes to the development of inclusive, interactive strategies that can enhance psychological well-being in educational settings.

Despite ongoing advances in pedagogy, the integration of emotional development within formal educational contexts remains limited. Socio-emotional learning (SEL) fosters skills like emotional regulation, empathy, decision-making, and interpersonal communication, which are crucial for students’ mental well-being and academic success (Immordino-Yang et al., 2019; Cipriano et al., 2020). These competencies are particularly essential in higher education settings, where students experience a critical developmental stage often marked by psychological and social challenges.

Recent research has also emphasized the relevance of socio-emotional learning (SEL) for specific student populations, such as individuals with Autism Spectrum Disorder (ASD), for whom the development of emotional understanding and social competence is a central challenge in both educational and social domains. Studies have shown that structured SEL interventions can significantly enhance emotional awareness, communication, and adaptive behaviors among students with ASD (Chaidi and Drigas, 2022).

Furthermore, the integration of immersive technologies, particularly virtual reality, has demonstrated promising results in improving social and emotional functioning in neurodiverse learners. For instance, VR-based interventions have been effective in supporting adolescents with developmental language disorders and autism by providing safe, interactive environments for practicing social interaction and emotional regulation (Yang et al., 2025; Arts et al., 2025). These findings highlight the potential of VR as an inclusive educational tool capable of addressing diverse emotional and cognitive profiles.

The COVID-19 pandemic intensified the urgency to address emotional dimensions in education, exposing vulnerabilities related to anxiety, stress, and lack of motivation among students in virtual environments. As noted by Dewa et al. (2024), the abrupt shift to remote learning disrupted students’ support networks and increased feelings of isolation, leading to greater demand for mental health interventions embedded within educational strategies. These challenges prompted calls for innovative methods that integrate technology with emotional skill-building to enhance student resilience (WHO, 2022).

Recent evidence supports the use of immersive technologies such as virtual reality (VR) to address these concerns. Aranda Romo and Caldera Montes (2019) demonstrated that gamified learning environments can promote integrative cognitive and emotional engagement, helping students internalize socio-emotional skills through interactive mechanisms. Moreover, studies by Sousa Ferreira et al. (2021) emphasize the utility of simulated scenarios for developing emotional competencies in a safe, controlled space.

Virtual reality offers unique affordances for socio-emotional learning, including immersive and personalized experiences that increase motivation and emotional connection to the content. According to Jensen and Konradsen (2018), VR can provide authentic and engaging simulations that allow learners to practice social and emotional behaviors in real-time, leading to deeper learning outcomes. Additionally, the emotional resonance of immersive experiences has been shown to support empathy, self-awareness, and communication skills (Colombo et al., 2021; Kyaw et al., 2019).

The aim of this exploratory quantitative study was to evaluate students’ acceptability of the VR scenarios, describe perceived safety indicators and immersion tolerance, and examine self-reported emotional, motivational, and socio-emotional responses using validated psychometric instruments. Expected outcomes included feasibility indicators (acceptability and usability), basic perceived safety, and descriptive patterns of emotional and motivational responses across VR scenarios.

Building on this perspective, the next section outlines the theoretical and empirical foundations that support the use of virtual reality as an educational tool for socio-emotional learning.

## Virtual reality

The term “virtual reality” (VR) was originally coined by Jaron Lanier in the 1980s. Today, VR is defined as a subfield of computer science that involves the design and implementation of immersive three-dimensional environments using computing devices, sensory input/output peripherals, and simulation software. These environments provide users with the sensation of being physically present in a simulated world, allowing interaction and engagement with virtual elements in real-time (Slater and Sanchez-Vives, 2016; Freeman et al., 2017).

Although VR is widely recognized for its entertainment applications—particularly in gaming—it has expanded into various sectors such as education, medicine, psychology, architecture, and marketing. In the field of psychology, VR has demonstrated potential in treating a range of mental health conditions, including phobias, anxiety, post-traumatic stress disorder (PTSD), and depression. One of the key advantages of VR-based therapy is the ability to simulate controlled, repeatable, and customizable environments for therapeutic exposure (Maples-Keller et al., 2017; Bell et al., 2020).

In educational contexts, VR can function as a transformative tool for experiential learning, allowing students to engage actively with content in ways that traditional methods cannot match. VR applications have been shown to improve emotional engagement, memory retention, and cognitive flexibility in learners (Radianti et al., 2020; Jensen and Konradsen, 2018). These benefits make VR a particularly promising approach for enhancing socio-emotional skills such as empathy, emotional regulation, and stress management.

Recent studies highlight VR’s effectiveness in promoting psychological well-being among students. For example, a study by Colombo et al. (2021) showed that immersive VR interventions targeting emotional regulation significantly reduced stress and improved classroom dynamics in higher education settings. Similarly, a meta-analysis by Kyaw et al. (2019) confirmed that VR-supported learning environments improve outcomes in both cognitive and affective learning domains.

Moreover, advances in affordable VR hardware and software—such as Oculus Quest or mobile VR apps—are facilitating the integration of immersive technologies into regular classroom settings (Pottle, 2019). These tools provide educators with accessible ways to address emotional development and mental health challenges among students.

Thus, virtual reality is not merely a technological novelty; it is a viable pedagogical and therapeutic instrument for working with socio-emotional dimensions in educational environments. When thoughtfully integrated, it can enrich the educational process and contribute to students’ holistic development.

## Mental health and socio-emotional skills

Mental health is a multidimensional concept that encompasses not only the absence of psychological disorders but also the presence of emotional well-being, resilience, and adaptive functioning in personal and social domains. Historically, definitions of mental health have evolved from purely clinical perspectives to more holistic approaches that integrate psychological, social, and environmental factors (Galderisi et al., 2015; WHO, 2022).

From a psychological standpoint, individuals’ emotional states play a crucial role in their mental health. These states are often influenced by contextual variables such as socioeconomic status, family dynamics, and access to supportive relationships. Emotional disturbances, including anxiety, depression, and stress, are increasingly prevalent among adolescents and university students, who are navigating complex developmental and academic challenges (Auerbach et al., 2018; Granieri et al., 2021).

In recent years, a growing body of research has emphasized the importance of emotional education as a preventative measure for mental health issues. Emotional education aims to develop emotional competencies—understood as a set of abilities related to emotional awareness, expression, regulation, and autonomy—as well as social skills and life competencies (Bisquerra, 2021). These competencies are associated with higher levels of psychological well-being, academic achievement, and interpersonal satisfaction (Domitrovich et al., 2017; Taylor et al., 2017).

Socio-emotional skills, often embedded within the broader framework of social and emotional learning (SEL), are defined as the cognitive and behavioral abilities that facilitate self-awareness, social awareness, relationship skills, responsible decision-making, and self-management (CASEL, 2023). These skills are critical for helping individuals adapt to diverse social contexts and manage emotional responses in constructive ways.

Numerous studies have highlighted the need to foster socio-emotional skills in educational contexts, especially given their protective role against emotional disorders. For instance, a longitudinal study by Jones et al. (2021) found that strong SEL development in adolescence predicted lower levels of psychological distress and better academic engagement in early adulthood. Similarly, interventions that target SEL have been shown to reduce symptoms of anxiety, depression, and aggression while enhancing positive emotionality and resilience (Murano et al., 2020).

Given this evidence, educational systems are increasingly called upon to integrate emotional education into curricula and daily practice. However, as several authors note, this integration requires a cultural and institutional shift that prioritizes emotional development alongside academic achievement (Schonert-Reichl, 2019; OECD, 2021). Technologies such as virtual reality present novel opportunities to support this integration through engaging, immersive environments that simulate real-life emotional challenges and facilitate experiential learning.

## Applications of virtual reality to mental health

Over the past two decades, educational institutions have increasingly explored digital technologies as part of broader innovation initiatives. Despite these efforts, the integration of such technologies often remains confined to disciplinary content areas, with limited application in fostering personal and emotional development. A common shortcoming in these initiatives is their continued reliance on traditional, teacher-centered methodologies, which do not align with the more dynamic, learner-centered frameworks required for the effective development of socio-emotional competencies.

Virtual reality (VR) has emerged as a promising tool in clinical psychology, particularly for its applications in diagnosing and treating mental health disorders. Unlike traditional interventions, VR enables patients to experience immersive, realistic scenarios that replicate everyday life while allowing for full environmental control by the therapist. This controlled immersion enables more effective management of phobias, social anxiety, PTSD, and emotional dysregulation (Valmaggia et al., 2016).

One of the key benefits of VR in therapeutic contexts is the capacity to customize exposure based on individual patient needs, which makes it ideal for cognitive-behavioral approaches (Freeman et al., 2017). Recent clinical trials show that VR-based therapy can produce significant reductions in anxiety symptoms and avoidance behaviors (Chard et al., 2023; Bisso et al., 2020). These outcomes are particularly relevant for adolescent and young adult populations, who may experience increased emotional vulnerability and stigma associated with traditional therapy formats.

In the educational field, VR has been implemented both as a therapeutic and pedagogical resource to strengthen self-regulation, attention, and motivation. For instance, Gutiérrez et al. (2019) found that primary education students training to become future teachers demonstrated high levels of motivation and engagement when using VR to explore historical content, illustrating the technology’s dual pedagogical and emotional value.

Palanques Alegre et al. (2022) conducted a study with adolescents undergoing therapeutic intervention for impulse control issues. Their experimental design compared traditional cognitive-behavioral therapy to the same approach enhanced with VR support. Results indicated significantly improved emotional regulation, focus, and verbal expression in the VR group, suggesting that immersive environments amplify the efficacy of behavioral interventions.

Similarly, Servera et al. (2020) explored the feasibility of using VR to address specific phobias such as fear of the dark among children. Their findings suggest that VR therapy can be implemented effectively even by non-expert facilitators, although further studies are needed to establish its long-term effectiveness.

In neuropsychology, VR-based tools are gaining attention for their potential in both assessment and intervention. Martínez Feu (2017) evaluated the AULA Nesplora test—a VR-based diagnostic tool designed to assess attention-deficit/hyperactivity disorder (ADHD). Results confirmed the tool’s capacity to improve the accuracy of psychopedagogical assessments while also enhancing user engagement.

Moreover, family-focused psychoeducational VR platforms, such as the Tagette software developed by Álvarez Cedillo et al. (2022), have demonstrated promise in improving understanding of schizophrenia among support networks. These tools help caregivers empathize with patients’ daily challenges, thus promoting more effective support and reducing emotional distress in caregivers themselves.

From a public health perspective, the SANABIEN VR project illustrates how VR can serve as a preventive mental health intervention. This mobile VR-based therapy encourages emotional expression among users, who later receive professional mental health support. Preliminary data indicate that such approaches can reduce therapy dropout rates and increase early intervention success (Cáceres Díaz et al., 2023).

Educational applications of VR have also expanded post-COVID-19, focusing on mitigating the psychological effects of physical isolation and enhancing learning quality. Studies by Sousa Ferreira et al. (2021) and Gómez-García et al. (2019) highlight the potential of VR in domains ranging from physical education to vocational training, where immersive learning positively impacts emotional engagement and personal development.

In higher education, VR has also been used to address complex social topics such as gender violence, empathy, and anxiety reduction. For instance, Rodero and Larrea (2022) implemented a VR-based training program to foster empathy and reduce anxiety among university students, showing positive emotional outcomes across experimental groups.

These findings collectively support the use of virtual reality as a multifaceted tool for enhancing mental health through both clinical and educational frameworks. When applied strategically, VR offers experiential, engaging interventions that align with contemporary understandings of emotional development, especially in youth populations.

## Materials and methods

### Study design

This study follows an applied research design with a descriptive exploratory methodological framework aimed at understanding the impact of virtual reality as a pedagogical strategy to develop socio-emotional competencies. The study employed a quantitative, descriptive, cross-sectional design. No qualitative methods, focus groups, recordings, or coding procedures were used. Data collection relied exclusively on validated self-report psychometric instruments. The design was chosen to address both the emotional needs of students and the potential of immersive virtual environments.

A literature review supported the theoretical framework and conceptual foundation, while primary data were collected using structured surveys and psychometric pretests administered remotely through digital platforms. The study adheres to ethical standards outlined by APA (2017) and obtained informed consent from all participants.

### Participants

This study was conceived as an exploratory pilot project aimed at identifying the emotional needs and behavioral patterns of first-year university students. The sample size (n = 14) was determined through purposive non-probabilistic sampling, based on accessibility and the suitability of participants for testing an immersive VR-based intervention in a real educational setting. Small-sample pilot studies have been recognized as valuable for refining instruments, methodologies, and intervention protocols (Leon et al., 2011; Thabane et al., 2010).

The study sample consisted of 14 first-year undergraduate students enrolled in a Computer Systems Engineering program at a public higher education institution in Sinaloa, Mexico. The sampling technique was non-probabilistic and purposive, based on participants’ willingness to participate and their potential to benefit from the proposed intervention. All participants signed an informed consent form and were informed of their right to withdraw from the study at any time without consequences.

### Instruments

Two structured self-report instruments were designed drawing on indicators established in psychological and educational research. The first was a structured survey to construct a digital psychological profile, which included six core areas: perceptual difficulties, experience with psychological care, manifestations of emotional disorders, family environment, emotional regulation scenarios, and school-related motivation.

The second instrument was a 19-item pretest designed to identify potential socio-emotional challenges, including anxiety, impulsivity, emotional dysregulation, and stress responses. The items were based on indicators from the Difficulties in Emotion Regulation Scale (DERS) and the Generalized Anxiety Disorder scale (GAD-7), both validated tools widely used in psychological research (Gratz and Roemer, 2004; Spitzer et al., 2006).

Both the DERS and GAD-7 were administered in their validated Spanish-language versions. The GAD-7 was scored by summing item responses (0–3) for a total score of 0–21, following established clinical cutoffs. The DERS was scored using standard Likert-based scoring to obtain subscale and total emotion-dysregulation scores. Analyses were performed using SPSS version 26.

### Virtual reality platform

After reviewing recent applications of immersive technology in educational and therapeutic contexts (Radianti et al., 2020; Jensen and Konradsen, 2018), the virtual reality platform Psious® was selected for its robust functionality and evidence-based applications in emotional training and mental health. Psious^®^ provides guided VR environments designed to address issues such as social anxiety, public speaking, stress management, and emotional regulation (Gutiérrez-Maldonado et al., 2016).

The platform was used to design personalized VR exposure sessions aligned with each participant’s needs. Sessions were planned based on data collected during the diagnostic phase and were implemented using virtual scenarios replicating school, family, and social environments.

### Ethical considerations

The study was approved by the institutional research ethics committee. Participants were informed of the objectives, procedures, and potential risks and benefits of the study. Confidentiality and anonymity were maintained throughout, in compliance with ethical research standards (APA, 2017; World Medical Association, 2013).

Although the study was not a clinical intervention and did not include therapeutic follow-up, all participants were informed about the availability of the university’s psychological counseling service. The institutional crisis-response protocol was available to any student reporting emotional distress or risk indicators. Participants could request immediate confidential support at any moment throughout or after the study.

Reports of suicidal ideation were obtained exclusively through self-report items and did not constitute a clinical diagnosis. In cases where participants indicated significant emotional discomfort, they were advised to contact institutional mental health services as part of the established university protocol.

## Results

The analysis of the initial survey and pretest instruments provided relevant insights into the socio-emotional conditions and psychological indicators present in the student sample.

### Psychological care experience

**Figure 1:**
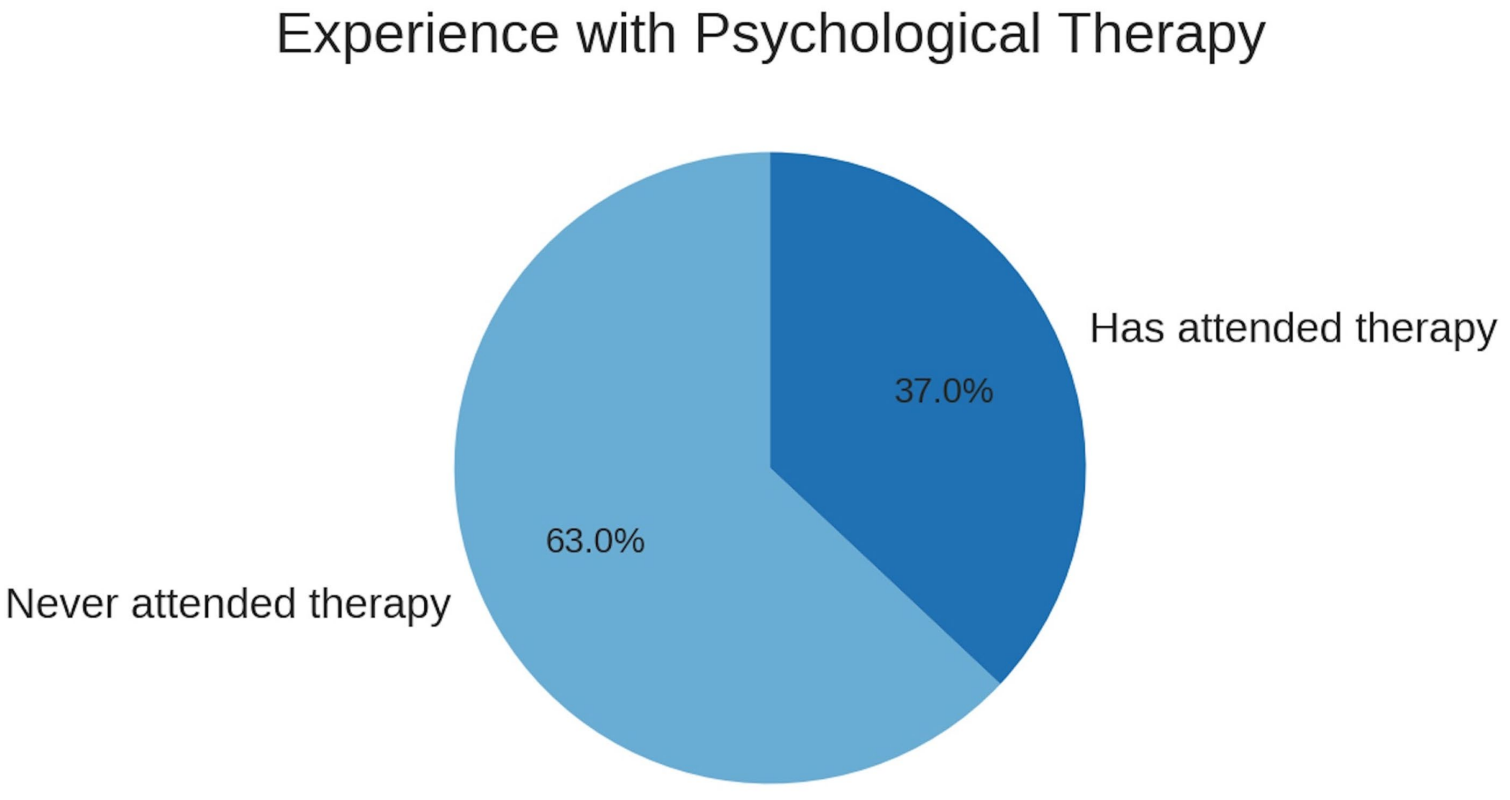
Self-reported prior experience with psychological therapy among participants. Percentages represent the proportion of students indicating previous attendance or no prior attendance.

A significant portion of participants (63%) reported never having attended psychological therapy, despite acknowledging signs of emotional distress (see Figure 1). This highlights a discrepancy between perceived needs and the actual use of mental health services, a phenomenon frequently observed in university populations (Granieri et al., 2021).

### Self-perception of mental health

**Figure 2:**
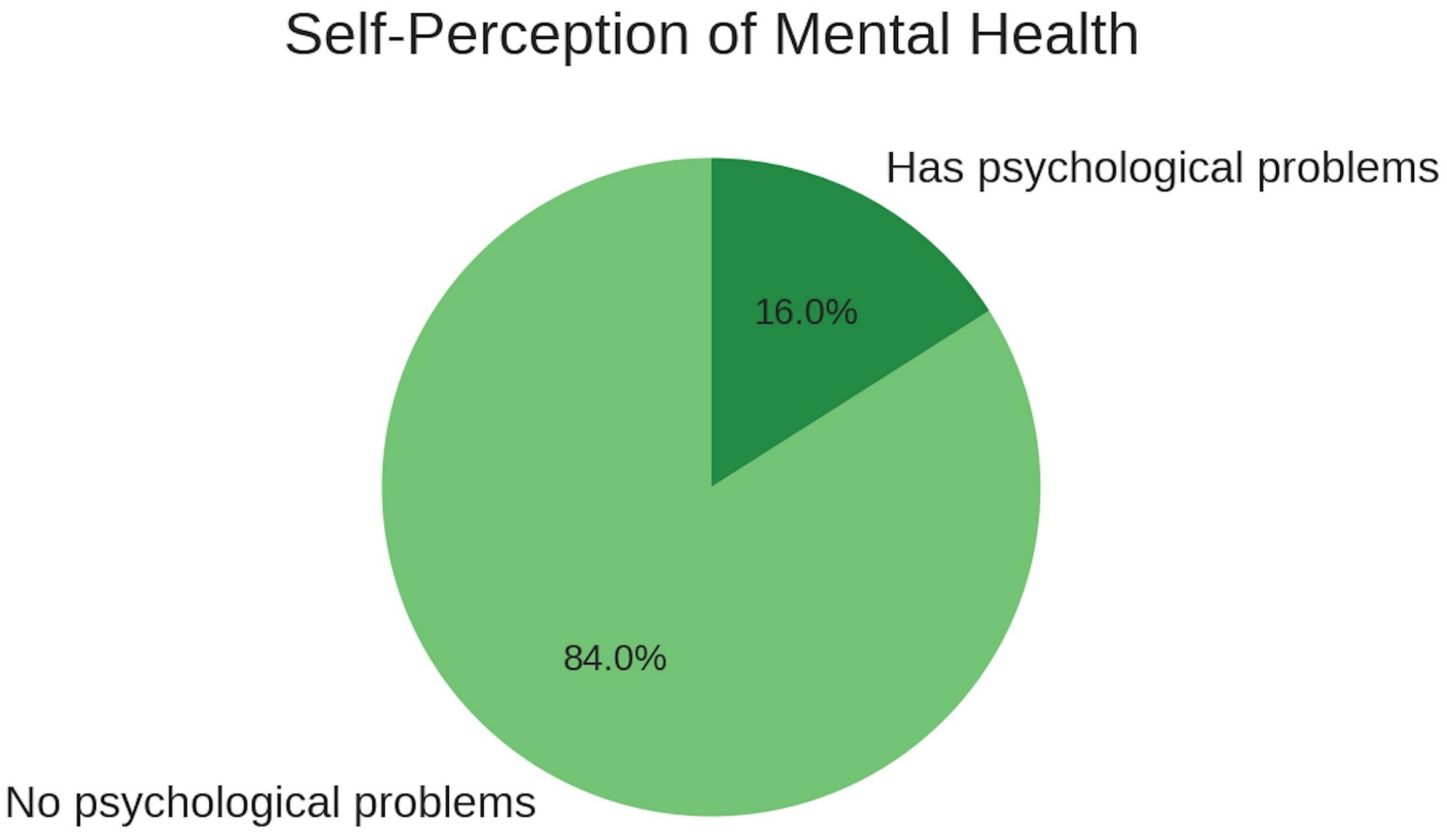
Participants’ self-perceived mental health status. Percentages indicate the proportion of students reporting the presence or absence of psychological problems.

In terms of mental health self-perception, 84% of students reported not having psychological problems, while 16% acknowledged some form of distress. Among this 16%, one-third identified anxiety, another third mentioned family and insecurity issues, and the remaining third referred to depressive or mixed symptoms (see Figure 2). These findings align with global studies indicating underreporting and normalization of emotional discomfort among university students (Dewa et al., 2024).

### Academic and personal performance factors

**Figure 3:**
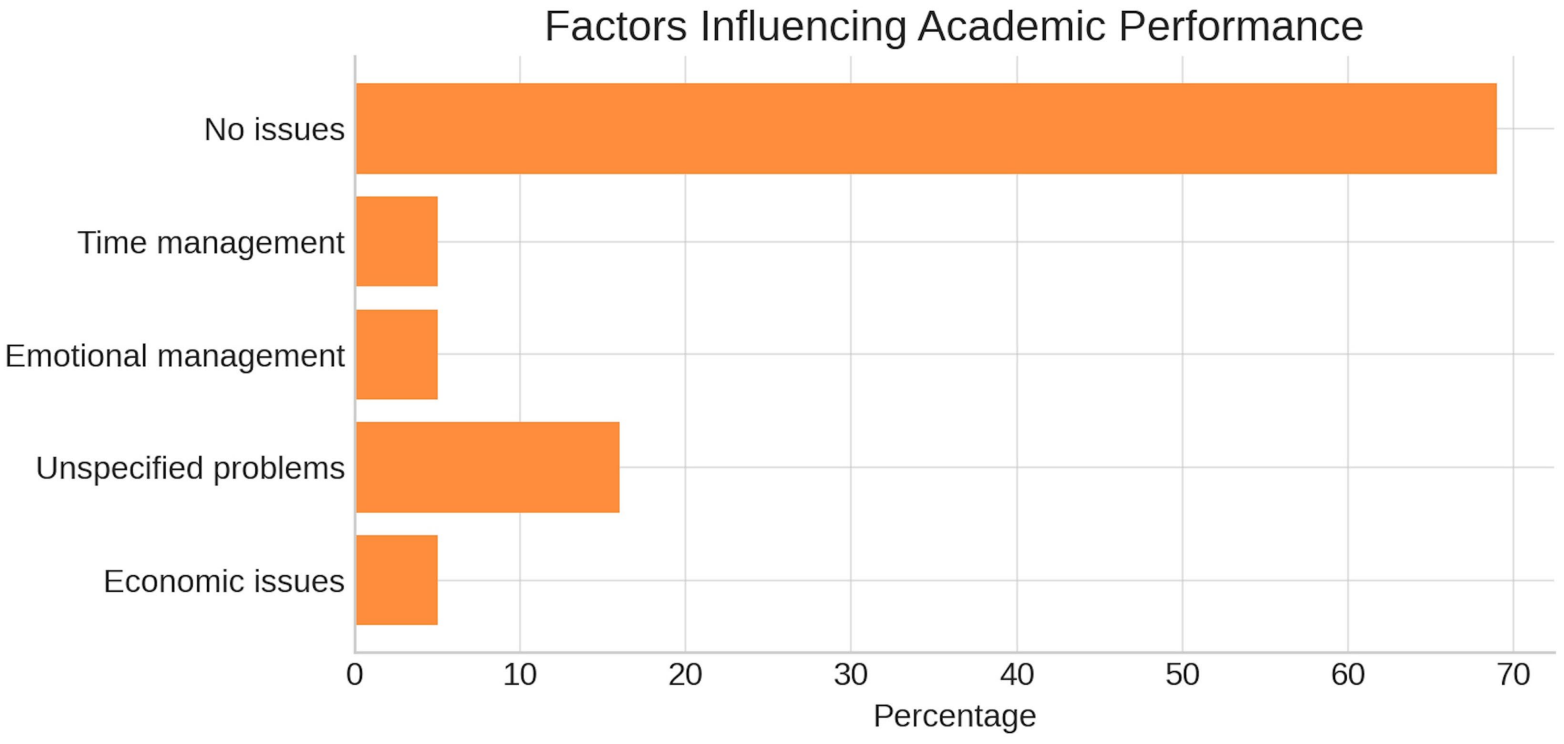
Self-reported factors influencing academic performance among participants. The figure displays the percentage distribution of students reporting different factors, including time management, emotional management, economic issues, unspecified problems, or no reported issues, based on survey responses.

Regarding elements that influence academic or personal performance, 69% of participants reported no significant issues. However, 16% disclosed unspecified problems, and others reported emotional regulation difficulties, time management issues, and financial constraints (see Figure 3).

### Family environment and communication

**Figure 4:**
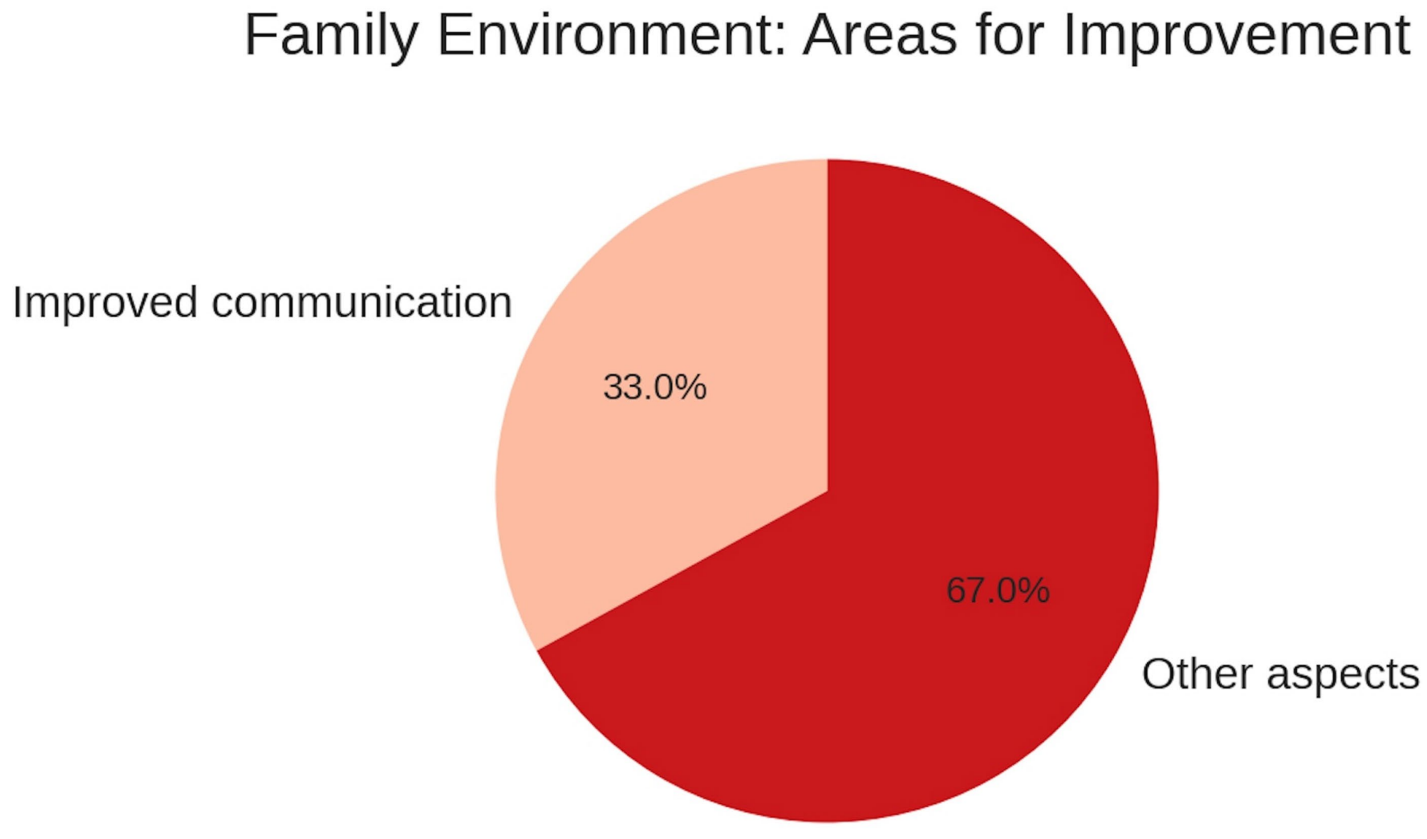
Percentage distribution of self-reported areas for improvement within the family environment among participants.

When asked about aspects of the family environment that could enhance academic performance, one-third of students highlighted the need to improve communication with their parental figures. This result suggests that family dynamics are seen as important for academic and emotional success, consistent with findings that emotional competence is influenced by familial emotional modeling and expressiveness (Peng et al., 2021) (see Figure 4).

### Academic motivation

**Figure 5:**
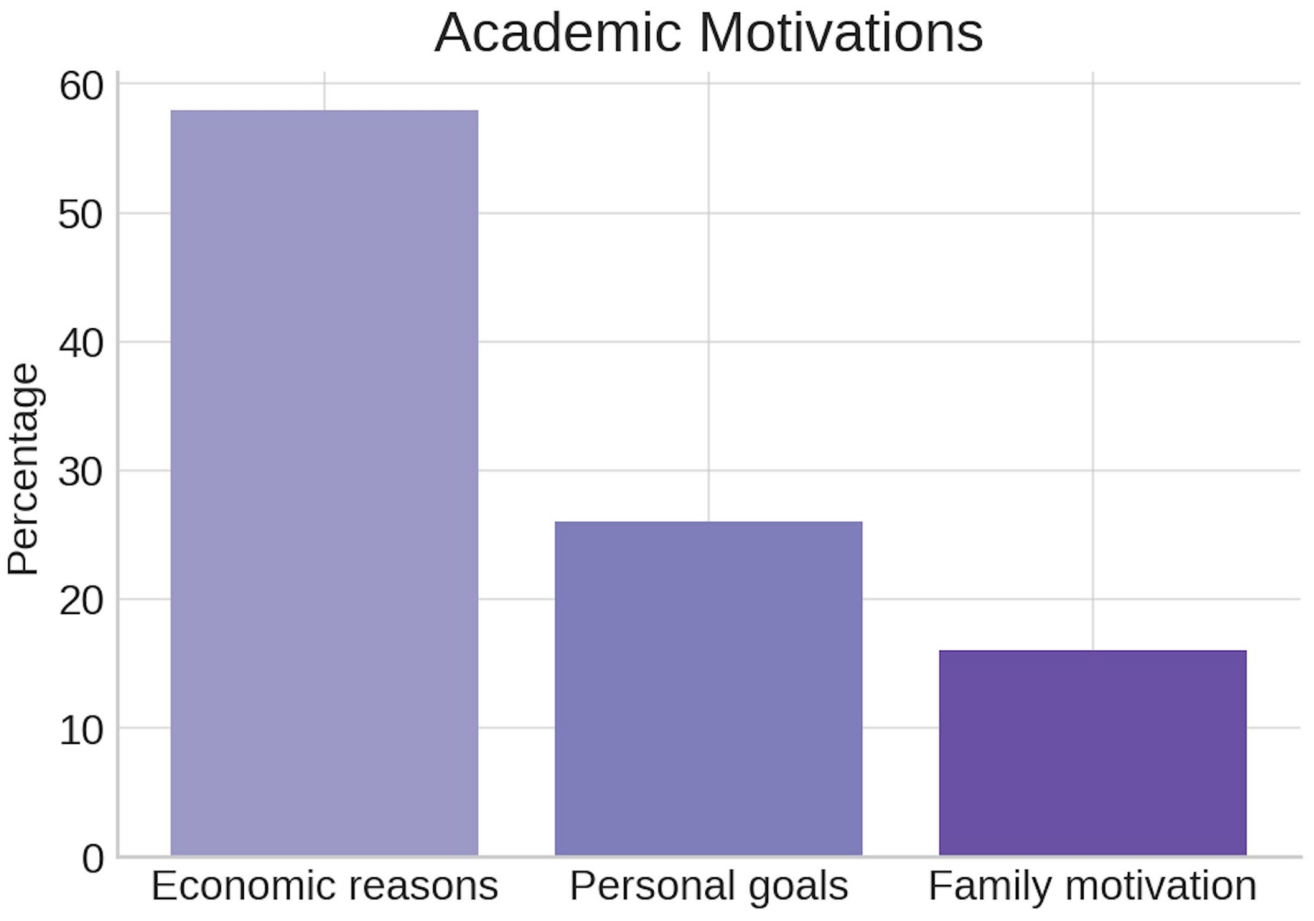
Percentage distribution of self-reported academic motivation factors among students.

Academic motivation was mostly attributed to economic reasons (58%), followed by personal aspirations (26%) and family expectations (16%). These motivational factors are considered central components in educational engagement models and may interact with emotional resilience and goal setting (Schunk and DiBenedetto, 2020) (see Figure 5).

### Emotional and behavioral indicators

In addition to the survey data, participants responded to a pretest assessing emotional and behavioral tendencies. Table 1 summarizes the life experiences reported by the students that may affect their mental health. Notably, sleep-related disturbances, anxiety symptoms, and psychosomatic expressions such as onychophagia and sweaty palms were commonly reported. Alarmingly, 26% of students disclosed experiencing suicidal thoughts, underscoring the urgency of intervention efforts in this population.

**Table 1:** Life situations experienced by participants that may impact mental health.

Experienced situation	Percentage (%)
Desire to sleep frequently	68%
Insomnia	58%
Nail biting (onychophagia)	47%
Nightmares	47%
Balance issues	37%
Visual hallucinations	32%
Dizziness or fainting	32%
Fears or phobias	32%
Sweaty hands	32%
Panic attacks	26%
Suicidal thoughts	26%
Accidents	21%
Stuttering	21%
Auditory hallucinations	16%
Substance use	16%
Head trauma	16%
Learning difficulties	16%
Nervous tics	11%

The variety and frequency of symptoms reported support existing research indicating that emotional dysregulation and somatic complaints are prevalent among youth, especially during transitional phases such as entry into higher education (WHO, 2022).

### Virtual reality scenarios and intervention design

Following the analysis of the socio-emotional profile of participants, tailored virtual reality (VR) intervention sessions were developed. These sessions were based on the results of the pretest, which identified specific emotional and behavioral challenges among students. The selected VR scenarios aimed to support the development of key socio-emotional skills, particularly in areas such as anxiety management, emotional regulation, social interaction, and family communication.

The use of immersive technologies in therapeutic education has shown increasing efficacy in promoting emotional competencies, particularly when designed in alignment with students’ lived experiences and psychological needs (Freeman et al., 2017; Colombo et al., 2021). Table 2 presents the mapping of specific emotional indicators identified in the pretest to the VR scenarios used in the intervention program.

**Table 2:** Emotional indicators and corresponding VR scenarios used for intervention.

Pretest indicator/emotional situation	Associated VR scenario	Skill targeted
Fear of public speaking	Speaking in front of an audience	Stress and anxiety management
Conflict in family relationships	Communication with a family member in a tense situation	Assertiveness and empathy
Difficulty managing frustration	Coping with failure in a simulated academic challenge	Emotional regulation
Feelings of isolation or loneliness	Attending a social gathering	Social engagement and interaction
Trouble with authority or classroom behavior	Classroom scenario with instructor feedback	Behavioral self-control
Generalized anxiety symptoms (e.g., sweaty palms)	Relaxation in a guided natural environment	Self-awareness and relaxation skills
Impulsivity or attention problems	Task-switching in a distracting environment	Focus and impulse control
Mood instability or sadness	Conversation with a supportive peer	Emotional expression and connection

### Platform and technical specifications

The VR platform Psious^®^ was selected due to its clinical-grade design and broad library of psychologically validated scenarios. It allows for the personalization of sessions based on cognitive-behavioral therapy (CBT) principles and includes physiological monitoring tools such as real-time heart rate and stress levels (Bell et al., 2020).

Each student participated in a sequence of 4 to 6 planned VR sessions, each lasting approximately 20–30 min, administered in a controlled educational setting. Scenarios were selected individually based on the participants’ pretest profiles and self-reported needs. The structure of the sessions followed a standardized sequence: (1) pre-immersion briefing, (2) guided VR session, and (3) post-session reflection and feedback.

### Theoretical and pedagogical framework

This intervention is grounded in experiential learning theory and socio-emotional learning (SEL) frameworks, particularly those emphasizing immersive, participatory methods that promote reflection and skill acquisition through direct engagement (Kolb, 2014; Taylor et al., 2017). Additionally, the alignment between technological immersion and emotional stimulation is a key factor in the effectiveness of VR-based emotional education (Radianti et al., 2020; Jensen and Konradsen, 2018).

By incorporating real-life contextual challenges into simulated VR experiences, students were able to develop emotional regulation strategies in a risk-free environment. This approach has been shown to improve students’ motivation, resilience, and self-efficacy (Kyaw et al., 2019; Slater and Sanchez-Vives, 2016).

## Discussion and conclusions

The findings of this study confirm the significant potential of virtual reality (VR) environments as educational tools for the development and reinforcement of socio-emotional competencies in higher education students. Emotional indicators such as anxiety, low frustration tolerance, impulsivity, and communication challenges were prevalent among the participants and were effectively addressed through carefully designed immersive interventions.

The high prevalence of symptoms related to emotional distress—such as insomnia, panic attacks, and suicidal ideation—highlights the critical importance of integrating emotional support systems into educational institutions. These results align with international studies reporting similar mental health trends in university populations, exacerbated by academic pressures, social transitions, and post-pandemic effects (Auerbach et al., 2018; Dewa et al., 2024).

Virtual reality, as demonstrated in this study, offers a controlled, safe, and engaging environment for students to confront emotionally challenging scenarios and practice regulation strategies. The personalized use of VR scenarios—aligned with pretest findings—allowed students to experience emotionally meaningful and contextually relevant simulations. This experiential approach is supported by the broader literature on immersive learning, which suggests that high-fidelity simulations can promote not only emotional engagement but also behavioral change and skill transfer (Slater and Sanchez-Vives, 2016; Kyaw et al., 2019).

The results also support the idea that socio-emotional learning should be considered a core component of educational curricula, rather than an ancillary or optional add-on. Institutions that neglect emotional development risk compromising students’ academic achievement, well-being, and social functioning (OECD, 2021; Taylor et al., 2017). Participants in this study clearly identified areas such as family communication, stress from public speaking, and emotional regulation as domains where support is needed, reinforcing the relevance of SEL-focused interventions.

Furthermore, students’ lack of prior psychological support, despite evident needs, points to potential barriers in access to mental health services or a lack of awareness. As previous research has shown, integrating emotional support into familiar environments—like schools—can normalize emotional development and reduce stigma (Schonert-Reichl, 2019).

This study adds to the growing body of evidence supporting VR’s effectiveness as a complementary educational and therapeutic tool. The structured intervention model combining pre-diagnostic assessment with immersive simulation and guided reflection offers a replicable framework for institutions seeking to enhance students’ socio-emotional resilience.

### Limitations and future directions

As an exploratory pilot study, the limited sample size should be interpreted in the context of early-stage intervention design. The results are not intended to be generalized but rather to inform the development of scalable methodologies and to evaluate the practical feasibility of immersive VR in emotional education. Nevertheless, the findings offer valuable preliminary evidence and highlight key variables to be addressed in future research.

Although the findings are promising, the study’s small sample size and focus on a single institution limit the generalizability of the results. Future research should expand to include diverse populations and educational contexts and employ longitudinal designs to assess the long-term impact of VR-based socio-emotional interventions.

Additionally, future studies should explore the integration of biometric feedback (e.g., heart rate variability, skin conductance) into VR scenarios to enhance personalization and therapeutic monitoring, a growing trend in affective computing and digital mental health (Bell et al., 2020).

Future studies should consider expanding the sample size and integrating biometric indicators (e.g., skin conductance, heart rate) to further assess the personalization and effectiveness of VR-based socio-emotional interventions.

### Conclusion

The use of virtual reality in educational settings holds immense potential for the promotion of emotional well-being and the development of socio-emotional competencies. This study demonstrated that immersive VR experiences can serve as a pedagogical and therapeutic strategy to support students facing emotional challenges. As the field continues to evolve, the integration of technology with human-centered approaches will be essential in building inclusive, emotionally intelligent learning environments capable of responding to the complex realities faced by today’s youth.

## Data Availability

The raw data supporting the conclusions of this article will be made available by the authors, without undue reservation, upon reasonable request.
